# Linc00441 interacts with DNMT1 to regulate RB1 gene methylation and expression in gastric cancer

**DOI:** 10.18632/oncotarget.23928

**Published:** 2018-01-03

**Authors:** Jianping Zhou, Jun Shi, Xingli Fu, Boneng Mao, Weimin Wang, Weiling Li, Gang Li, Sujun Zhou

**Affiliations:** ^1^ Department of General Surgery, Yixing People’s Hospital The Affiliated Hospital of Jiangsu University, Yixing 214200, Jiangsu, P.R China; ^2^ Health Science Center, Jiangsu University, Zhenjiang 212000, Jiangsu, P.R China; ^3^ Department of Gastroenterology, Yixing People’s Hospital The Affiliated Hospital of Jiangsu University, Yixing 214200, Jiangsu, P.R China; ^4^ Department of Oncology, Yixing People’s Hospital The Affiliated Hospital of Jiangsu University, Yixing 214200, Jiangsu, P.R China; ^5^ Yixing People’s Hospital The Affiliated Hospital of Jiangsu University, Yixing 214200, Jiangsu, P.R China

**Keywords:** gastric cancer, linc00441, RB1, DNMT1, proliferation

## Abstract

Recent studies revealed that several Long noncoding RNAs (LncRNAs) are associated with progression of gastric cancer (GC), while the functional role and molecular mechanism of many GC-associated lncRNAs remain undetermined. The tumor suppressor-gene retinoblastoma gene (RB1) was decreased in several human cancers including gastric cancer (GC). In this study, we investigated whether Linc00441 was involved in the suppression of RB1. Our findings showed that the up-regulated Linc00441 was inversely correlated with RB1 expression in human GC tumor samples. The gain- and loss-of-function investigation revealed that Linc00441 could promote the proliferation of GC cells. Furthermore, RNA pull down and RIP assays demonstrated that Linc00441 could recruit DNMT1 to the RB1 promoter and suppressed RB1 expression in GC cells. In conclusion, our findings revealed that Linc00441 played crucial role in GC progression and suggested that Linc00441 was potentially an effective target for GC therapy in the future.

## INTRODUCTION

Gastric cancer (GC) is the third leading cause of cancer death worldwide. GC is one of the prominent cancers with high morbidity and mortality in developing countries and half of the world total occurs in eastern Asia (mainly in China). The rate of GC has dropped greatly over the last half-century in most developed western countries, but has not decreased in East Asia [1, 2]. Deeper understanding of the molecular mechanism of GC will provide novel insights into its pathogenesis. Recently, long noncoding RNA (lncRNA) is emerging as new players in disease biology [1]. However, the roles of lncRNAs in GC are still an emerging field need to be investigated.

The long non-coding RNAs (lncRNAs that are > 200 nt long), that are coded by the genome but are mostly not translated into proteins, play key roles in regulating gene expression, chromatin dynamics, differentiation, growth, and development [3, 4]. LncRNAs are a novel class of potential biomarkers and therapeutic targets for the treatment of cancer [5]. In recent years, a growing number of cancer transcriptomes has been examined through next-generation sequencing, and we indeed find thousands of lncRNAs whose aberrant expression is associated with different cancer types. However, several lncRNAs have been linked to malignant transformation, only few have been functionally characterized [6]. LncRNAs are expressed in a regulated manner and have tissue-specific expression. LncRNAs can influence pluripotency, immune response, cell survival, or cell cycle among other functions, which determine the transformed phenotype of cancer cells. Additionally, several lncRNAs are transcriptionally regulated by key oncogenes (c-myc) or tumor suppressors (p53) [7–9].

The retinoblastoma susceptibility gene (RB1), the tumor suppressor gene, is mutated at variable frequencies in numerous human cancers. In addition, RB1 is a negative regulator of cell proliferation and a chromatin-associated protein that limits the transcription of cell cycle genes [10]. Primarily, RB1 restricts the expression of genes that are necessary for cell proliferation via suppressing transcription of E2F targets [11]. Moreover, RB1 was down-regulated in gastric cancer [12, 13].

In this study, we elucidate the impact of lncRNA Linc00441 in linking RB1 and gastric cancer. Linc00441 was found up-regulated and inversely correlated with RB1 expression in gastric cancer. Its function in GC cells as a tumor promoter and the related mechanisms are studied. The results indicate that Linc00441 is a potential suppressor of RB1 via binding DNMT1 to RB1 promoter thus to promote cell proliferation in human GC.

## RESULTS

### Linc00441 was up-regulated and inversely correlated with RB1 expression in gastric cancer

**Figure 1:**
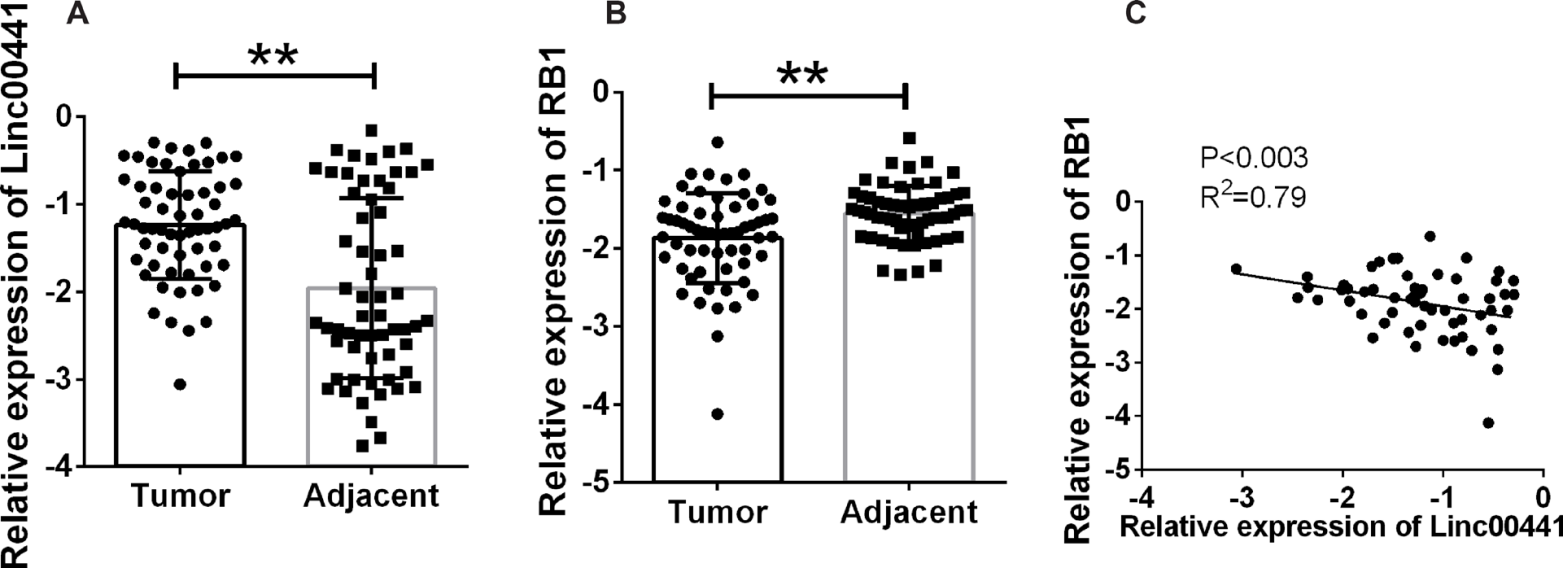
Relative expression of Linc00441 and RB1 in patients with gastric cancer (**A**) A relatively increased level of Linc00441 was detected in GC tissues compared with the corresponding adjacent tissues (*n* = 70). (**B**) The decreased level of RB1 was detected in GC tissues compared with the corresponding adjacent tissues (*n* = 70). (**C**) Pearson correlation showed a positive correlation between expression levels of RB1 and Linc00441 with a *P* < 0.003, R^2^ = 0.79. Data was presented as the mean ± SEM. Data was presented with median and interquartile range by log-transformed. Wilcoxon rank sum test was applied for statistical analysis. ^**^ indicated *p* < 0.01.

To determine whether Linc00441 was involved in the pathogenesis of GC, we performed the real-time PCR in 70 pairs GC tumor tissues and corresponding adjacent tissues and found that the expression of Linc00441 was increased in human GC tumor tissues (Figure 1A). We also found that the expression of was decreased in tumor tissues (Figure 1B). To further investigate the association between Linc00441and RB1 expression in gastric cancer, we tested the correlation between them using Pearson’s correlation analysis, and identified a strong inverse correlation between Linc00441 and RB1 expression (Figure 1C) (*P* < 0.003 and R^2^ = 0.79).

### The long non-cording RNA Linc00441 located in the nuclear

**Figure 2:**
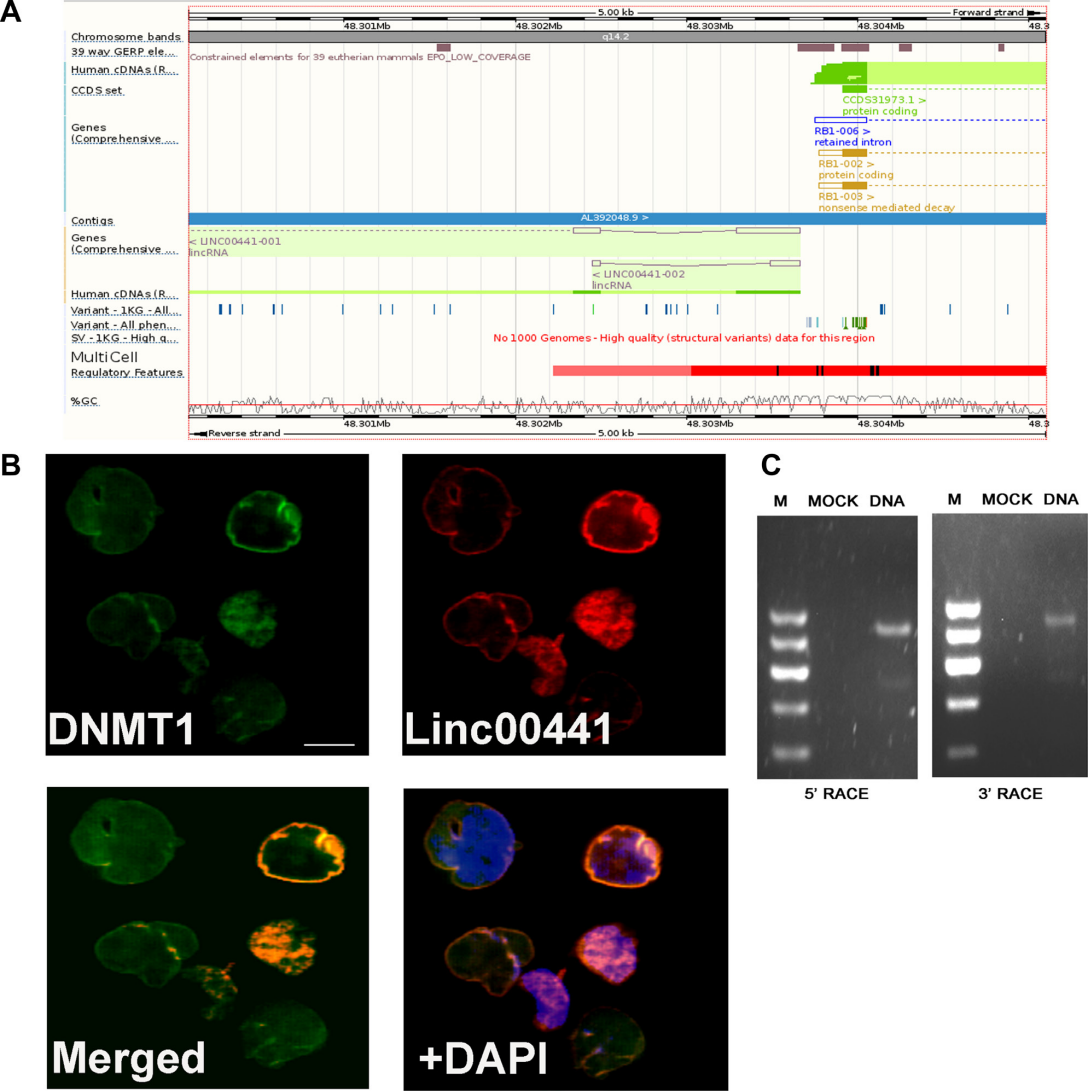
The characteristic of Linc00441 (**A**) The relative physical location of Linc00441 and RB1 obtained from PubMed database. (**B**) The subcellular location of Linc00441 was detected by using probe targeting Linc00441 in GC paraffin sections and the location of DNMT1 was detected by immunofluorescence. (**C**) 5’RACE PCR and 3’RACE PCR was used to detected the full length of Linc00441 in cells.

The relative physical genomic location of Linc00441 and RB1, which obtained from PubMed database, was presented in Figure 2A. We next analyzed the subcellular location of Linc00441 by using probe-targeting Linc00441 in AGS cells and found that Linc00441 located in the nuclear (Figure 2B). The biologic function of Linc00441 in gastric cancer cell remains unclear. Therefore, 5′and 3′ RACE analysis were performed to determine the full-length Linc00441 (Figure 2C). Taken together, these data demonstrated that Linc00441 is a long non-cording RNA and located in the nuclear.

### The effect of Linc00441 on the regulation of gastric cancer cell proliferation

**Figure 3:**
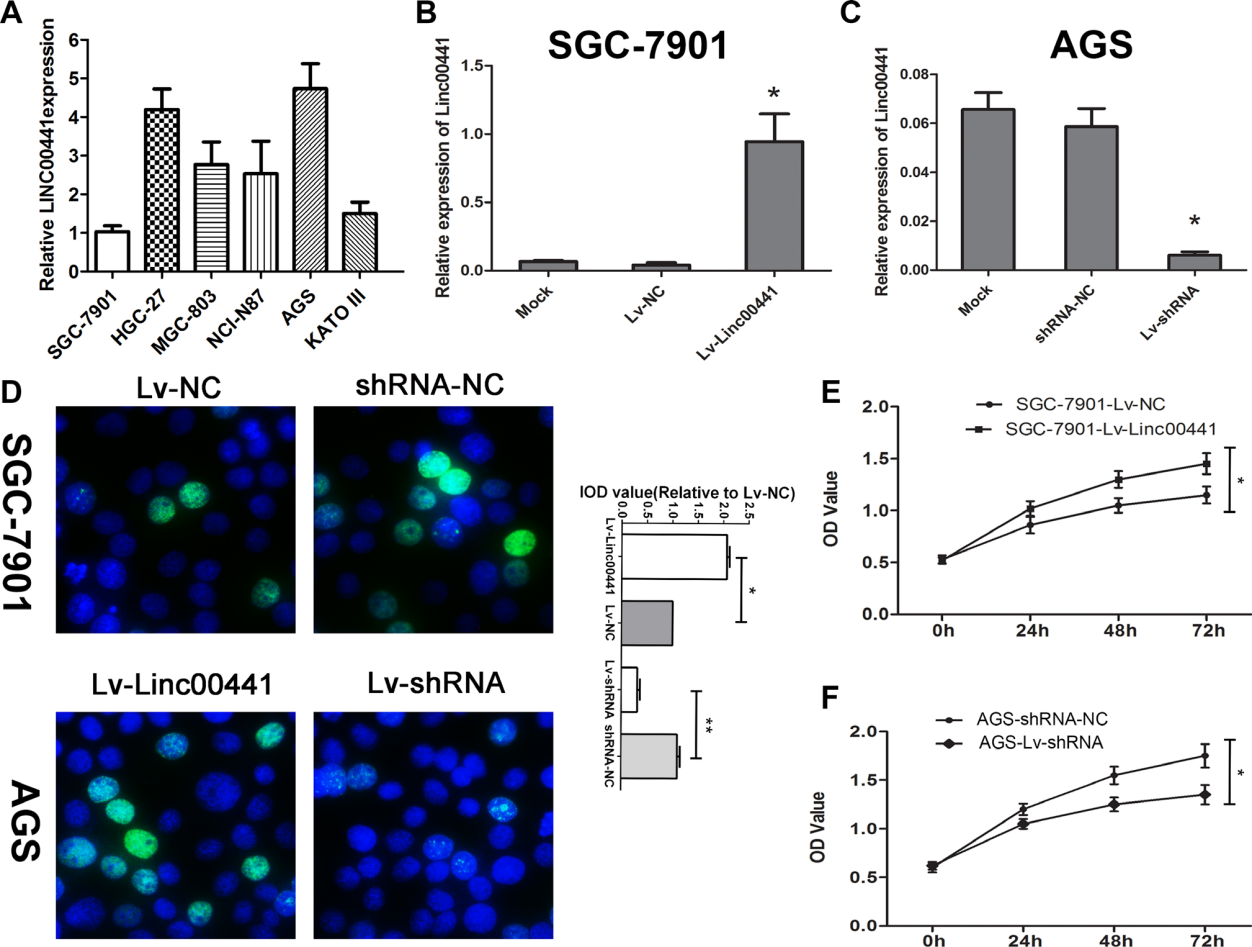
Linc00441 promoted cell proliferation *in vitro* (**A**) Relative expression of Linc00441 in different GC cell lines. (**B**) The relative expression of Linc00441 in SGC-7901 cells treating with Linc00441 overexpression lentivirus. (**C**) The relative expression of Linc00441 in AGS cells treating with Linc00441 shRNA. (**D**) The EDU assay confirmed the functional role of Linc00441 in cell proliferation. (**E** and **F**) The CCK8 assay presented showed that a decreased level of Linc00441 inhibited the growth of GC cells. Absorbance at 450 nm was presented as the mean ± SEM. The integral optical density value of cells treated with control plasmids was normalized to 100%. All experiments were performed in triplicate and presented as the mean ± SEM. ^*^ indicates a significant difference compared with the control group (*P* < 0.05).

To further study the association between Linc00441 expression and gastric cancer, we tested Linc00441 expression in various human gastric cancer cell lines and found that Linc00441 was up-regulated in HGC-27 and AGS cells and down-regulated in SGC-7901 and KATO III cells compared with MGC-803 and NCI-N87 cells (Figure 3A). To investigate the function of Linc00441 in gastric cancer cells, we carried out gain- and loss-of-function experiments respectively by introducing either the Linc00441 overexpression or Linc00441 shRNA lentivirus for Linc00441 into gastric cancer cells. Then, we performed knockdown of Linc00441 in AGS cells that with higher Linc00441 expression and up-regulated its expression in SGC-7901 cells that with low Linc00441 expression (Figure 3B and 3C). The EDU assays showed that silencing Linc00441 significantly reduced cell proliferation in AGS cells and increasing Linc00441 significantly enhanced cell proliferation in SGC-7901 cells (Figure 3D). To confirm the role of Linc00441 in gastric cancer cell proliferation, we further examined cell proliferation ability by the CCK8 assay and found that knockdown of Linc00441 impaired cell proliferation, while Linc00441 over-expression promoted cell proliferation *in vitro* (Figure 3E and 3F).

### Linc00441 suppressed RB1 expression in gastric cancer cells

**Figure 4:**
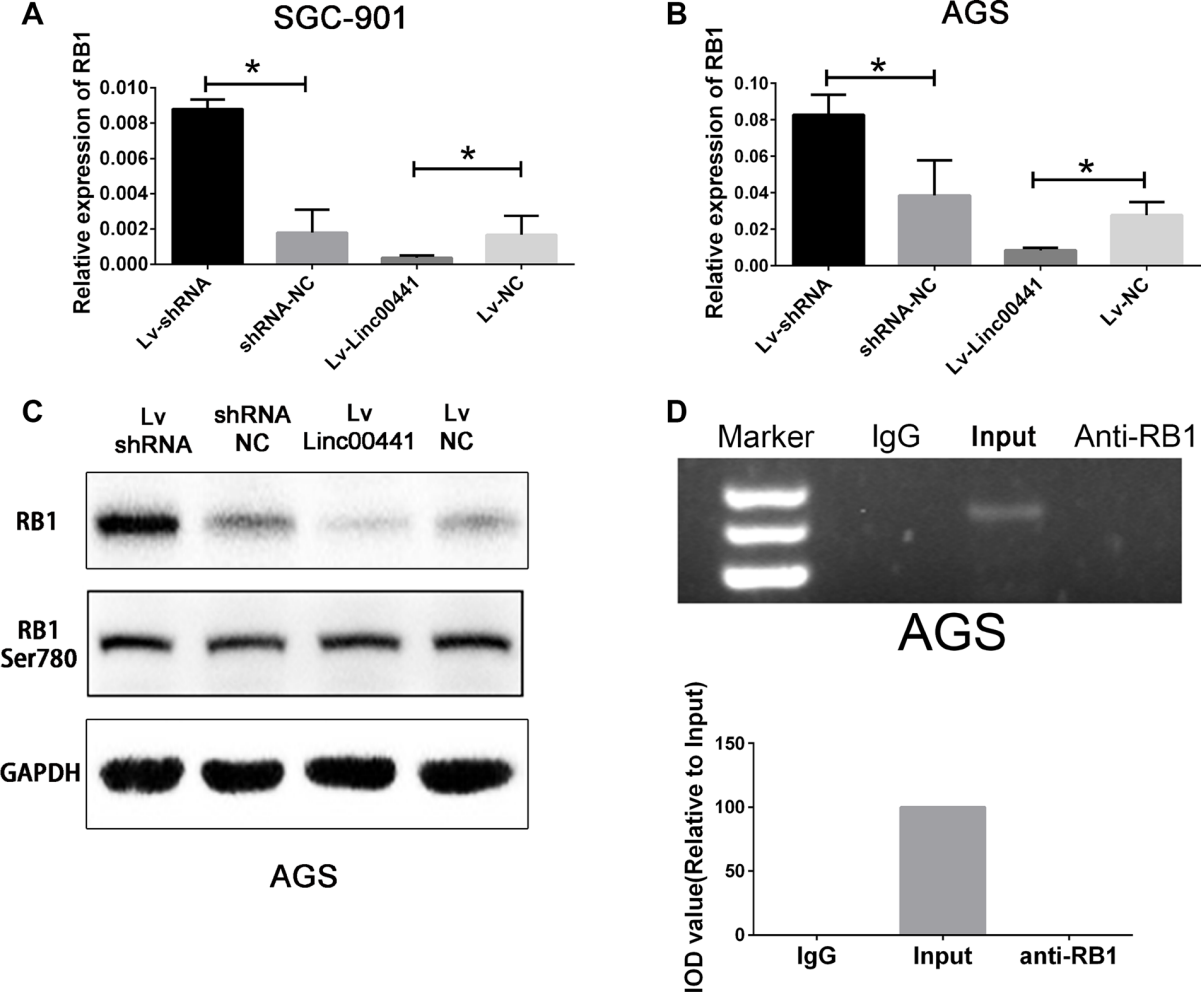
Linc00441 could suppress RB1 expression (**A**) and (**B**) The relative expression of RB1 in cells treating with Linc00441 overexpression or shRNA lentivirus. (**C**) The protein expression of RB1 and phosphorylated RB1 in cells treating with Linc00441 overexpression or shRNA lentivirus. (**D**) Relative RIP experiments were performed using an antibody against RB1 on extracts from AGS with IgG as a negative control. The enrichment of the Linc00441 was normalized to the input. The purified RNA was used for RT-PCR analysis. The results showed that no bands were detected from the RNA in the group with anti-RB1.

To investigate whether RB1 expression is suppressed by Linc00441, we performed real-time PCR and Western blotting assays. The results showed that ectopic expression of Linc00441 dramatically abrogated the expression level of RB1 mRNA in both AGS and SGC-7901. Moreover, RB1 mRNA level was substantially elevated in the both AGS and SGC-7901 with Linc00441 knock-down (Figure 4A and 4B). The Western blotting results showed that RB1 protein increased in the AGS-shRNA cells and decreased in the AGS-Linc00441 cells (Figure 4C). However, no change in the level of RB1 phosphorylation was detected (Figure 4C). Our results indicated that Linc00441 might bind and suppressed RB1 in GC cells. To verify this hypothesis, the Linc00441 over-expression lentivirus was infected into AGS cells and we performed the RIP assays to examine the association between Linc00441 and RB1. However, the RIP assays in AGS cells infected with Linc00441 over-expression lentivirus showed that Linc00441 could not bind to RB1 (Figure 4D).

### Linc00441 could recruit DNMT1 to the RB1 promoter

**Figure 5:**
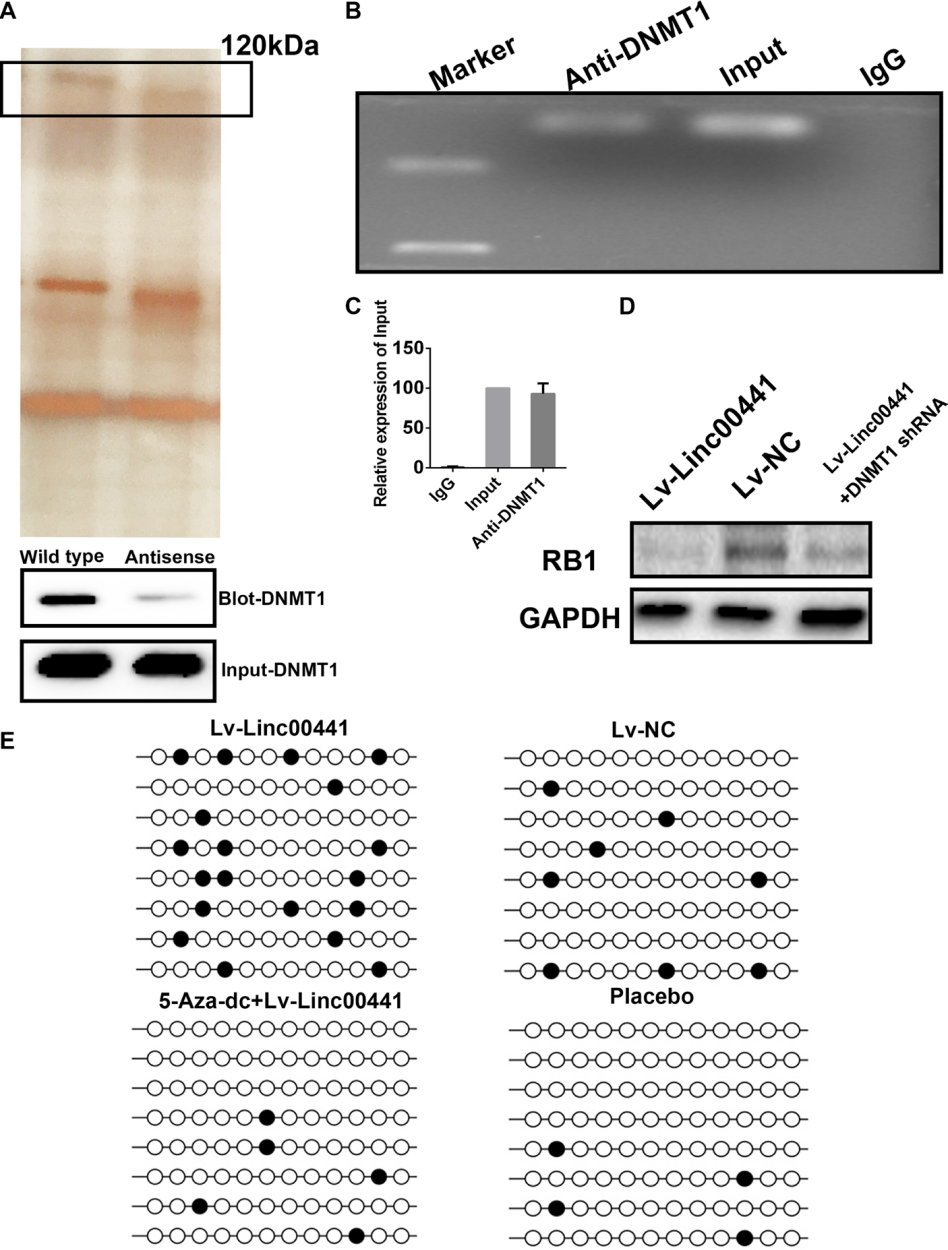
Linc00441 could induce RB1 methylation by recruiting DNMT1 (**A**) RNA pull-down assay of AGS extract from the different groups. The arrow indicated the band at 120kDa approximately. (**B**) RIP assay was performed using an anti-DNMT1 antibody and was confirmed with agarose gel electrophoresis using a different probe. (**C**) Fold increases were calculated by comparison with the input in the lower panel. (**D**) The DNMT1 was suppressed by using shRNA technology. After confirming the decreasing of DNMT1 in cells overexpressed with Linc00441, The expression of RB1 was detected by WB. (**E**) The DNA methylation rate was detected by bisulphite sequencing.

In order to discover the mechanism how Linc00441 can regulate the neighbor gene RB1. The RNA pull-down assay was further conducted. At first, the biotin-labeled lncRNA and the antisense as control was used followed by mass spectrum validation with protein band collected at the ∼ 120 kDa location. We have validate the specific interaction between DNMT1 and Linc00441 by employing the immunoblot assay. Antibody targeting RB1 was applied in the pulled protein lysate and the input protein. Through the mass spectrum screening, we found that peptide DNMT1 was most significant (Figure 5A). Thus, Linc00441 might bind with DNMT1 in GC cells. Moreover, we performed RIP assays in AGS cells infected with the Linc00441 over-expression lentivirus and confirmed that Linc00441 directly bind to DNMT1 (Figure 5B and 5C). The DNMT1 was further suppressed by using shRNA technology. After confirming the decreasing of DNMT1 in cells overexpressed with Linc00441, we investigated the expression of RB1, as presented in Figure 5D, the loss of DNMT1 was attenuated. The methylation patterns of RB1 gene promoter region in AGS-Linc00441 cells were analyzed using bisulphite sequencing. In theAGS-Linc00441cell, a decreased DNA methylation rate was found in the promoter of RB1 (Figure 5E).

## DISCUSSION

Human genome contains close to 16,000 genes that encode more than 28,000 distinct lncRNA transcripts according to the Encyclopedia of DNA Elements (ENCODE) Project Consortium (GENCODE release 23) [6]. Increasing studies have revealed that lncRNAs have important influence in tumorigenesis, metastasis, and prognosis and drug resistance of gastric cancer [1]. While the functional role and molecular mechanism of many GC-associated lncRNAs remain undetermined, recent studies revealed that several lncRNAs are associated with GC progression, such as H19, FENDRR, and MRUL [14–16]. In the present study, we showed that lncRNA Linc00441 was over-expression in GC. We further validated the Linc00441 expression was inversely correlated with RB1 expression in GC.

LncRNAs contribute to the hallmarks of cancer and are becoming attractive potential therapeutic targets [17]. Our findings also showed that knockdown of Linc00441 inhibited GC cell proliferation. The results suggested that Linc00441 might function as an oncogenic lncRNA in GC. Recent studies revealed that several lncRNAs are associated with tumorigenesis of human cancer [18]. However, the molecular mechanisms involved in lncRNAs functional role in cancer cells are not well known. In this study, our findings demonstrated that Linc00441 could suppressed RB1 expression in GC cells.

Studies have suggested some nuclear lncRNAs can antagonize the function of transfactor or chromatin complexes. For example, the oncogenic lncRNA SCHLAP1 antagonizes the regulatory functions of the SWI/SNF chromatin remodeling complex [19]. The tumor suppressor lncRNA *GAS5* binds to the DNA-binding domain of the glucocorticoid receptor (GR) and competes with DNA glucocorticoid response element (GREs) [20]. Our findings indicated that Linc00441 might bind to RB1 in GC cells. To verify this hypothesis, we performed the RIP assays to examine the association between Linc00441 and RB1. However, our results showed that Linc00441 did not directly bind to RB1. RB1, a tumor suppressor gene, is normally inactivated in numerous cancers. Researchers have revealed that the pRb protein acts a pivotal part in cell cycle regulation, predominantly during the G1-S transition [21]. When cells are stimulated by growth stimulation signals, the induced Cyclin D/CDK4 can contribute to phosphorylation of RB1, which leads to the release of E2Fs from RB1-E2Fs complex and S-phase entry [22]. The inactivation of an upstream RB regulator (CDNKN2a/INK4a/p16) selectively occurred in the RB positive tumors resulting in the RB/p16 pathway inactivation [23]. We have known that p16/p14 is involved in the regulation of cyclin D/CDK4 activity. Therefore, the elevation of cyclin D/CDK4 activity leading to Rb1 phosphorylation and E2Fs accumulation [24].

Moreover, tumor suppressor gene Rb1 promoter became hypermethylated due to DNMT1 overexpression in several human cancers [25–28]. DNMT1 maintains genomic DNA methylation at 5’-CpG-3’ residues in somatic cells [29]. DNMTs are overexpressed in GC. DNMT1 protein predominantly locates in the cytoplasm and nuclei in cells and is involved in GC progression [30, 31]. Thus, we hypothesis that lower expression of RB1 may be due to promoter hypermethylation of RB1 gene involved in the recruitment of DNMT1 by Linc00441. Here, RNA pull down and RIP assays demastrated that Linc00441 could bind to DNMT1 in GC cells (Figure 5). As well as our findings, Sun et al. have found that lncRNA HOXA11-AS stimulates cell proliferation and metastasis of GC via scaffolding the chromatin modification factors PRC2, LSD1 and DNMT1 [32]. Wang et al. have found that lncRNA Dum inhibits Dppa2, its adjacent gene, in cis by recruiting DNMT1, DNMT3A and DNMT3B [33]. In accordance, we found that increased expression of Linc00441 could enhance the methylation rate in the promoter region of RB1, which could be reversed by DNMTs antagonist 5-aza-2’-deoxycytidine (Figure 5D). In this study, we found that Linc00441 could recruit DNMT1 to the RB1 promoter and suppressed RB1 expression in gastric cancer cells.

Our results discovered that the lncRNA Linc00441 was an oncogenic lncRNA that promoted GC tumorigenesis through recruiting DNMT1 to target RB1 gene, leading to RB1 down-regulated and GC cell proliferation. Our findings revealed that Linc00441played crucial role in GC progression and suggested that Linc00441was potentially an effective target for GC therapy.

## MATERIALS AND METHODS

### Clinical samples

The clinical data and samples were obtained from 70 cases of patients who underwent gastric cancer radical resection surgery during July 2011 to October 2015 at Yixing People’s Hospital Affiliated to Jiangsu University (Wuxi, China). Patients had written informed consent. This study was approved by the Ethical Committee of Jiangsu University

### Cell culture and Ientiviral packaging

The cell lines were obtained from the American Type Culture Collection (ATCC) (Manassas, USA) and were maintained in 5% CO_2_ and DMEM with 10% fetal bovine serum (Gibico, USA). The full-length of Linc00441 or shRNA fragment were purchased from Genscript Co. Ltd. (Nanjing, China) and the sequences were sub-cloned into pLV-His or pLV-Luc plasmids. The palsmids were constructed for lentiviral packaging. In brief, the plasmids were co-transfected with dR8.91 and VSV-G in 293T cells. After culture for 72 hours, the medium supernatant was collected. Lentivirus particles was concentrated through ultracentrifugation.

### Real-time PCR

The expression levels of Linc00441 and other mRNAs were determined by using real time polymerase chain reaction (RT-PCR). The extraction of total RNAs was using TRIzol reagent according to the manufacturer’s protocol (Invitrogen, USA). RT-PCR was performed by using ABI Stepone Plus (Applied Biosystems, USA). The 2^-ΔΔCT^ method was used to calculate the expression of mRNAs. GAPDH was used as an internal control.

### Western blot

Proteins were extracted from tissues or cells by using the RIPA buffer containing PMSF (Beyotime, China). The same amount of protein loading in each lane was determined by the GAPDH. Antibodies including DNMT1 (ab13537), RB1 (ab24), p-RB1(Ser780) (ab47763) were purchased from Abcam (Cambridge, UK). After using the secondary antibodies (Santa Cruz, USA), the signals were detected by SuperSignal Chemiluminescent Substrate kit (Pierce, USA) according to manufacturer’s instructions. The integrated density of the band was confirmed by the Image J software (NIH, Bethesda, USA).

### Cell proliferation assay

The cell proliferation assay was performed by using CCK-8 (Dojin, Japan) and EDU (Millipore, USA) according to the manufacturer’s instructions. The cells were seeded at a density of 1 × 10^4^ cells/well in 96-well flat-bottom and respectively cultured for CCK-8 and EDU assays. CCK-8 value was measured at 0, 24, 48 and 72 hours. After culture for 48 hours, EDU assay was performed by using fluorescence microscope.

### RNA pull-down

The lncRNA was firstly labelled with biotin and then transcribed with a Biotin RNA Labeling Mix (Roche, CA, USA) along with the T7 RNA polymerase (Roche, CA, USA). Then, the products were treated with RNase-free DNase I (Roche, CA, USA) and purified with an RNeasy Mini Kit (Qiagen, Hilden, Germany). Proteins were incubated with biotinylated RNA. Then, the streptavidin agarose beads (Invitrogen, CA, USA) was added to each binding reaction. After wash for 5 times, the associated proteins were resolved by SDS-PAGE, and specific bands were excised and analyzed by mass spectrometry. Proteins in bands were eluted and digested. Digests were analyzed by Orbitrap Velos Pro LC/MS system (Thermo Scientific, CA, USA). Data was analyzed by Proteome Discoverer and the resulting peak lists were used for searching the NCBI protein database with the Mascot search engine.

### RNA immunoprecipitation assay (RIP)

RIP assay was performed by using EZ-Magna RIP™ RNA-Binding Protein Immunoprecipitation Kit (Millipore, MA, USA) according to the manufacturer’s instructions. The DNMT1 antibody was used for RIP (Abcam, Cambridge, UK). The co-precipitated RNAs were detected by reverse transcription PCR and real-time PCR. Total RNAs (input controls) and IgG were assayed simultaneously to establish that the detected signals were the result of RNAs specifically binding to DNMT1. The optical density was scanned by Image J and normalized to input group.

### Immunofluorescence

Fluorescence In Situ Hybridization (FISH) was performed by employing the FISH kit from Boster (Wuhan, China) according to the manufacturer’s instructions. Cells were fixed and permeablized using xylenes, ethanol and protease to allow biotin-labeled probes to access. Slides were treated with 30% H_2_O_2_ and ddH_2_O with the ratio of 1: 10 for 5 min, and then the 3% citric acid diluted pepsase was applied to expose the fragment of nucleic acid for 20 sec. The second fixation was followed by using 1% paraformaldehyde/0.1M PBS. Next, the slides were incubated with pre-hybridization solution at 40°C for 2 hours and then with lncRNA target probesat 30°C overnight followed by 2 washes with 2× saline sodium citrate (SSC). For immunofluorescence analysis, AGS cells were stained with rabbit anti-human DNMT1 followed by staining with Alexa Fluor 555-conjugated anti-rabbit IgG (Abcam, Cambridge, UK) antibodies. Positive cells were quantified using Image-Pro Plus software (Media Cybernetics, MD, USA) and detected by confocal microscopy (Zeiss, Oberkochen, Germany).

### DNA methylation

Genomic DNA was extracted from AGS cells treated with the Linc00441 expression lentivirus, 5-aza-2’-deoxycytidine and their control group cells. After adding sodium bisulfate from the EZ DNA Methylation-Gold™ Kit (Qiagen, Valencia, Canada)into the DNA samples, we tested the methylation in the promoter of RB1 using the method of bisulfite sequencing PCR.

### Statistical methods

All experiments were independently repeated at least triplicate. Data were expressed as mean ± SEM. Differences between two independent groups were tested with the student t test. All statistical analyses were carried out using SPSS version 18.0 and presented with Graphpad prism software. Pearson correlation analysis was performed in calculating the correlation. The results were considered to be statistically significant at *P* < 0.05.
